# Supplementary material for: Integrative transcription start site analysis and physiological phenotyping reveal torpor-specific expression program in mouse skeletal muscle
Source: Commun Biol. 2021 Nov 15;4:1290. doi: 10.1038/s42003-021-02819-2 (PMC8592991; doi:10.1038/s42003-021-02819-2)
Supplement: Supplementary file 1 — Supplementary Information [file 42003_2021_2819_MOESM1_ESM.pdf]

Supplementary Information

Integrative transcription start site analysis and physiological phenotyping reveal torpor-specific expression program in mouse skeletal muscle

Ruslan Deviatiiarov[1, 2], Kiyomi Ishikawa[3], Guzel Gazizova[1], Takaya Abe[4], Hiroshi Kiyonari[4], Masayo Takahashi[3], Oleg Gusev[1, 2, 5, 6*] and Genshiro A. Sunagawa[3*]

[1] Extreme Biology Laboratory, Institute of Fundamental Medicine and Biology, Kazan Federal University, Volkova str.18, Kazan, Tatarstan, 420008, Russian Federation.

[2] Endocrinology Research Center, Dmitriya Ul'yanova str. 11, 115478 Moscow, Russian Federation.

[3] Laboratory for Retinal Regeneration, RIKEN Center for Biosystems Dynamics Research, 2-2-3 Minatojimaminami-machi, Chuo-ku, Kobe, Hyogo, 650-0047, Japan.

[4] Laboratory for Animal Resources and Genetic Engineering, RIKEN Center for Biosystems Dynamics Research, 2-2-3 Minatojimaminami-machi, Chuo-ku, Kobe, Hyogo, 650-0047, Japan.

[5] Department of Regulatory Transcriptomics for Medical Genetic Diagnostics, Graduate School of Medicine, Juntendo University, Tokyo 113-8421, Japan.

[6] RIKEN Center for Integrative Medical Sciences, RIKEN, 351-0198 Yokohama, Japan.

* Corresponding authors: genshiro.sunagawa@riken.jp; o.gusev.fo@juntendo.ac.jp

Supplementary Figure 1, Fasting-induced Torpor Shows a Reversible Transcriptome Signature.

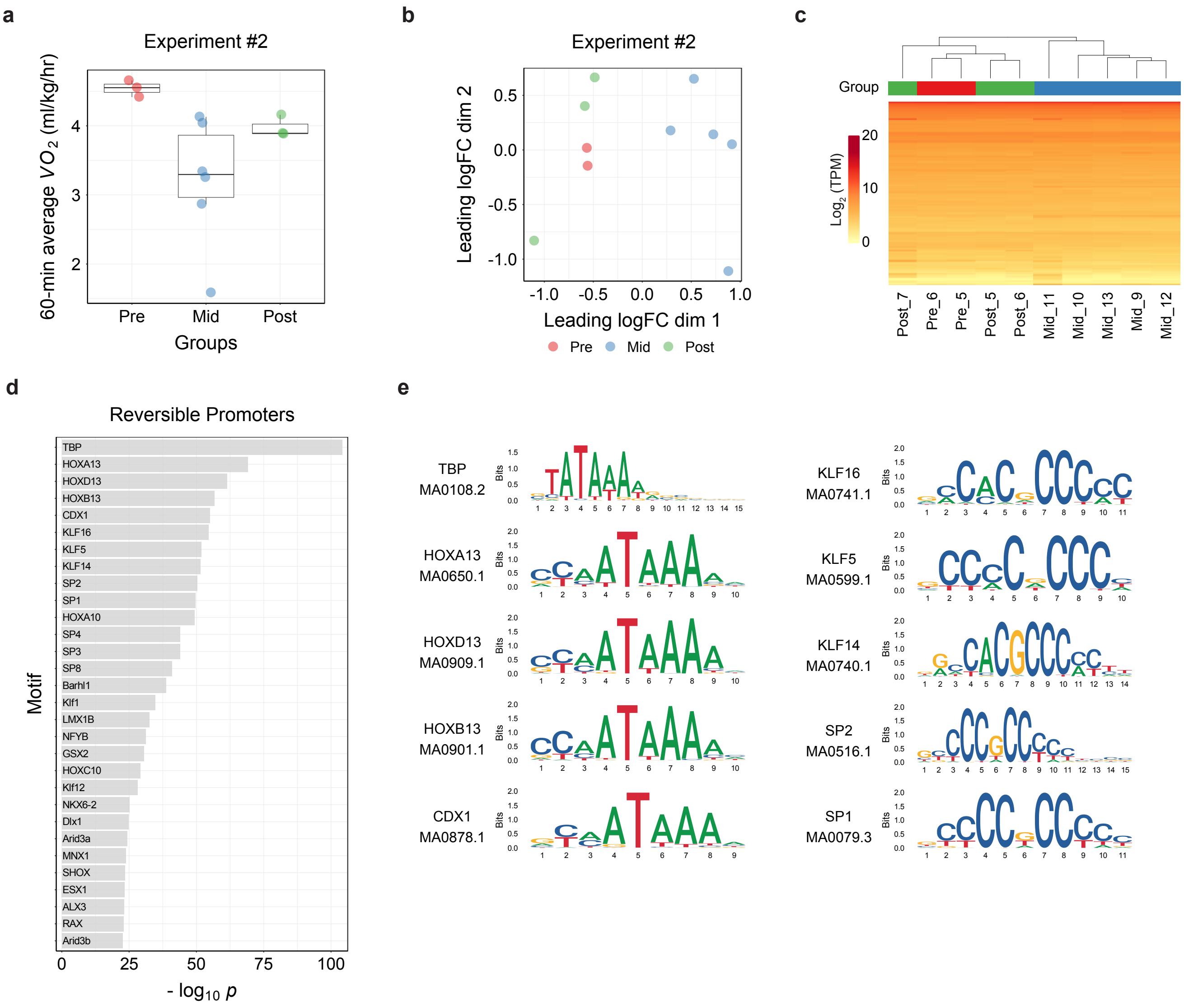

a Boxplots for the VO2 of animals at sampling in reversibility experiment #2. Each dot represents one sample from one animal. The results resembled the metabolic phenotypes as detected in experiment #1. See Fig. 1b. **b** MDS plot of the TSS-based distance in reversibility experiment #2. Each dot represents one sample from one animal. Note that the Mid group was clustered differently from the Pre and Post groups in the 1st dimension, as it were in Fig. 1c. **c** Hierarchical clustering heatmap based on the TPM of TSS detected in the reversibility experiment #2. The group colors are denoted in (b). **d** The top thirty motifs enriched in the reversible promoters. **e** Logos of the top ten motifs in the reversible promoters.

Supplementary Figure 2, Torpor Prevention at High T_A Revealed Hypometabolism-associated Promoters.

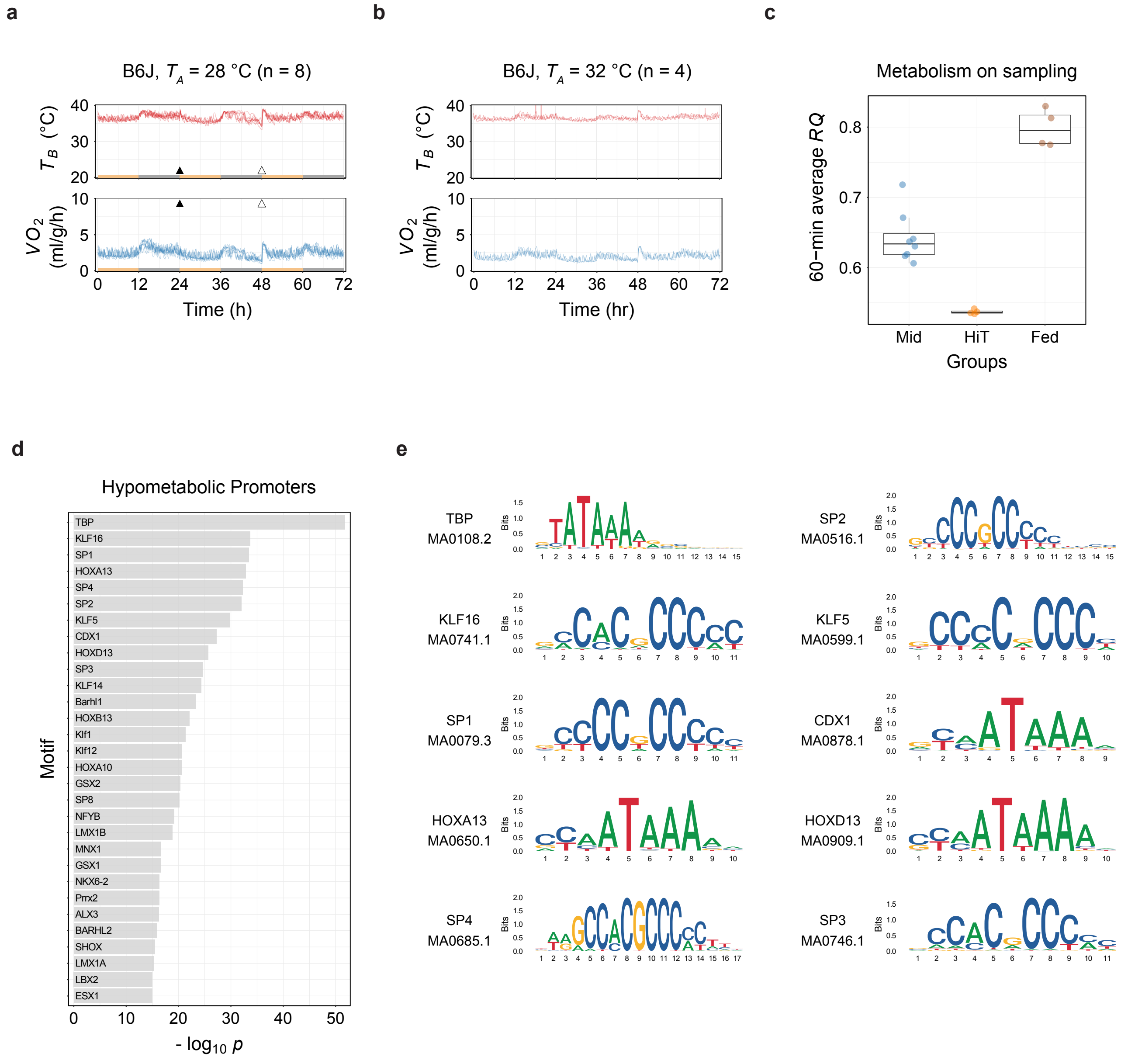

a Traces of T_B (red lines) and VO_2 (blues lines) of the B6J male mouse at T_A = 28 °C. **b** At T_A = 32 °C, no mouse entered torpor (n = 4). **c** Boxplots for the RQ of animals at sampling in the hypometabolic experiment. Each dot represents one sample from one animal. **d** Top thirty motifs enriched in the hypometabolic promoters. **e** Logos of the top ten motifs enriched in the hypometabolic promoters.

Supplementary Figure 3, Identification of Torpor-specific Promoters and their Dynamics.

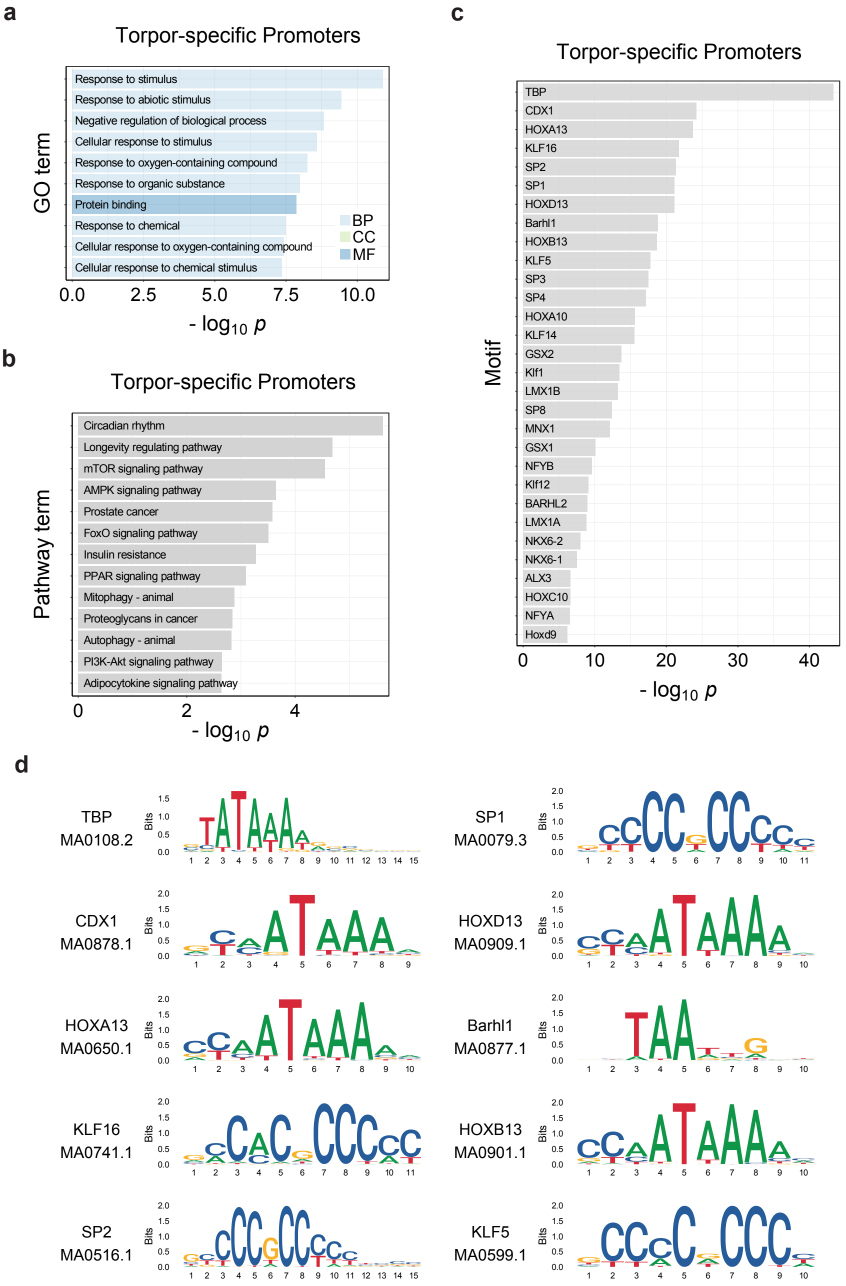

a Top ten enriched GO terms in the torpor specific promoters. **b** Of the 13 enriched KEGG pathways, the "mTOR signaling pathway" is shown as a representative example. Green and red denote up- and down-regulated genes, respectively. **c** Top thirty motifs enriched in the torpor-specific promoters. **d** Logos of the top ten enriched motifs in the torpor-specific promoters.

Supplementary Figure 4, *Atf3* is related to FIT regulation.

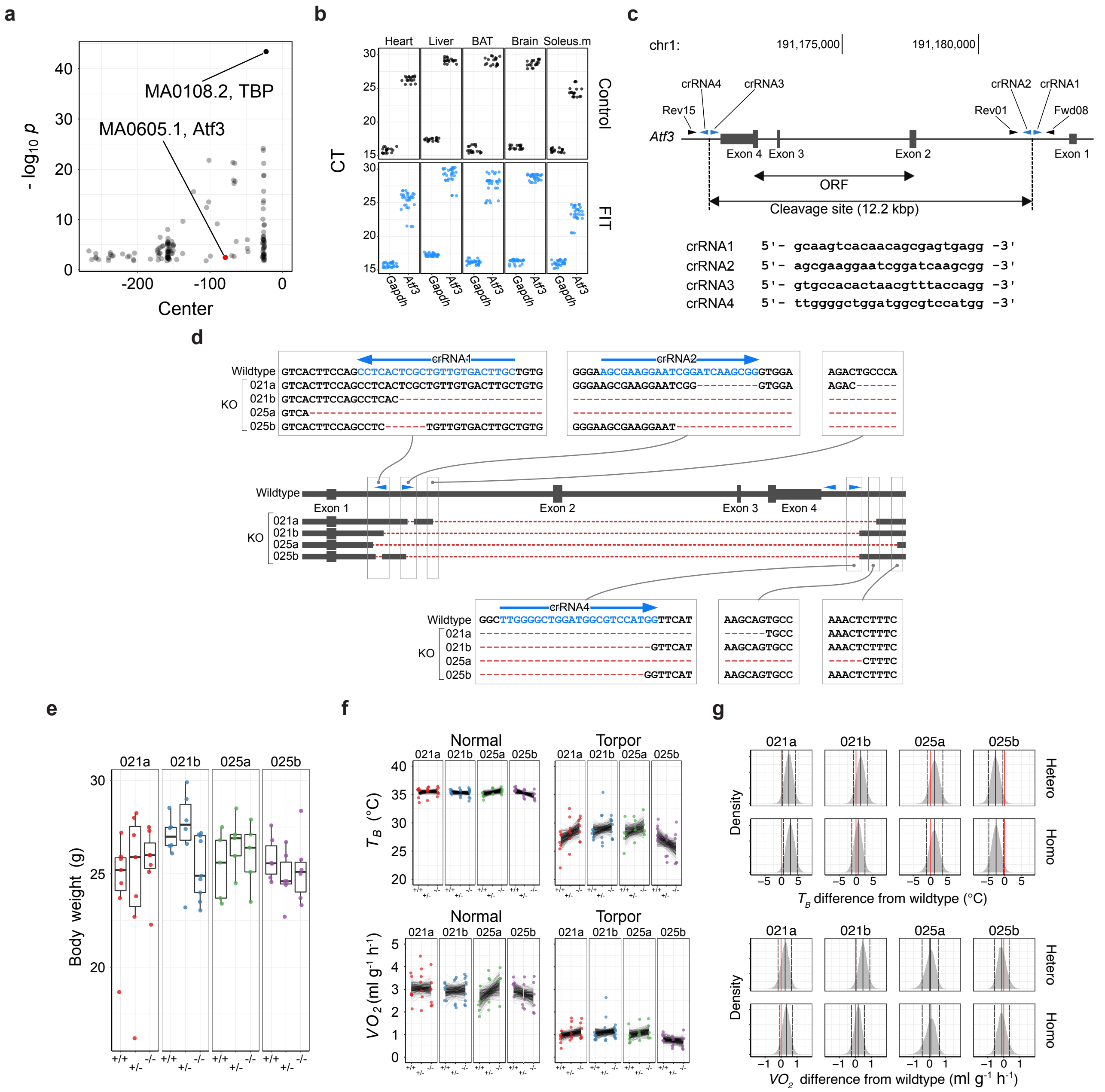

a Distribution of motifs enriched in the torpor-specific promoters. The horizontal axis denotes the position of the motif density peak from the TSS. The vertical axis denotes the p-value of the enriched motif. **b** Raw CT counts for mRNA quantification during torpor. ΔCT was calculated by the CT difference among *Atf3* and *Gapdh*, and ΔΔCT was calculated by the ΔCT difference among FIT and Control. **c** Schematic representation of the CRISPR/Cas9 gRNA sites (blue arrowheads) for the *Atf3*-KO generation as well as the genotyping primer sites (black arrowheads). **d** Deletion junction sequences for the four *Atf3*-KO alleles. All KO alleles had complete deletion of exon 2, 3, and 4. Blue arrow and arrowheads denote the gRNA site. Red dashed lines are the deleted sites. **e** The body weight of all *Atf3*-KO mice. **f** Minimal T_B and VO_2 of all *Atf3*-KO mice. **g** The difference of minimal T_B and VO_2 from wildtypes were estimated for heterozygous and homozygous KO strains. The solid vertical line shows the median of the posterior distribution, and the dashed lines show the lower and higher 89% HPDI ranges. The red line denotes zero for each box. T_B of ATF3-021a was estimated to have the 89% HPDI range larger than zero, which can be interpreted as both hetero and homo KO mice have higher T_B than wildtype animals. T_B of ATF3-025b was estimated to have lower T_B than wildtype, which is the opposite phenotype of the original *Atf3*-KO-025.
