# Supplementary material for: Integrative transcription start site analysis and physiological phenotyping reveal torpor-specific expression program in mouse skeletal muscle
Source: Commun Biol. 2021 Nov 15;4:1290. doi: 10.1038/s42003-021-02819-2 (PMC8592991; doi:10.1038/s42003-021-02819-2)
Supplement: Supplementary file 4 — Description of Additional Supplementary Files [file 42003_2021_2819_MOESM4_ESM.pdf]

Description of Additional Supplementary Files

File name: Supplementary Data 1

Description: Table of Differentially Expressed Promoters During Torpor.
